# Patterns and predictors of early mortality among emergency department patients in Addis Ababa, Ethiopia

**DOI:** 10.1186/s13104-015-1592-z

**Published:** 2015-10-24

**Authors:** Cheryl Hunchak, Sisay Teklu, Nazanin Meshkat, Christopher Meaney, Lisa Puchalski Ritchie

**Affiliations:** Department of Family and Community Medicine, Schwartz/Reisman Emergency Medicine Institute, Mount Sinai Hospital, University of Toronto, 600 University Avenue Rm 206, Toronto, ON M5G 1X5 Canada; Department of Obstetrics and Gynecology and Emergency Medicine, Addis Ababa University School of Medicine, Zambia Street, Addis Ababa, Ethiopia; Department of Medicine, University of Toronto, University Health Network, 200 Elizabeth Street, Toronto, ON M5G 2C4 Canada; Department of Family and Community Medicine, University of Toronto, 500 University Avenue, Toronto, ON M5G 1V7 Canada

**Keywords:** Emergency medicine, All-cause mortality, Ethiopia, Predictors, Mortality, Global health

## Abstract

**Background:**

Ethiopian emergency department (ED) patients have a considerable burden of illness and injury for which all-cause mortality rates have not previously been published. This study sought to characterize the burden of and to identify predictors for early all-cause mortality among patients presenting to the Tikur Anbessa Specialized Hospital ED (TASH-ED) in Ethiopia.

**Methods:**

Data was prospectively collected from the records of all patients who died within 72 h of ED presentation. Pearson’s Chi square and Fisher’s exact tests were used to investigate associations between two outcome variables: (a) time to death and (b) immediate cause of death in relation to specific demographic and clinical factors. Time from ED presentation to death was dichotomized as ‘very early’ mortality within ≤6 h and death >6–72 h and logistic regression was used to assess the adjusted impact of these demographic and clinical variables on the probability of dying within 6 h of ED presentation.

**Results:**

Between October 2012 and May 2013, 9956 patients visited the ED and 220 patients died within 72 h of admission. After excluding patients dead on arrival (n = 34), the average age of death was 43.1 years and the overall mortality rate was 1.9 %. Head injury (21.5 %) and sepsis (18.8 %) were the most common causes of death. Relative to medical patients, trauma patients were more likely to be male (p < 0.01), less likely to have had prior recent ED visits (p < 0.01) and more likely to be triaged as higher acuity (p = 0.04). The sole statistically significant predictor of death within 6 h from our multivariable logistic regression model was symptom duration less than 4 h (4–48 h vs. <4 h: OR = 0.20, 95 % CI 0.07, 0.53, p < 0.01; >48 h vs. <4 h: OR = 0.27, 95 % CI 0.09, 0.81, p = 0.02).

**Conclusions:**

The mortality burden of trauma and sepsis in the TASH-ED is substantial, and mortality patterns differ between these groups. As emergency medicine develops as a specialty in the Ethiopian health system, the potential impact of context-specific clinical care protocol development, trauma prevention advocacy and ED care re-organization initiatives to reduce mortality among these young, previously well patients warrants exploration.

## Background

Mortality from traumatic injuries and medical illness among Ethiopian emergency department (ED) patients remains largely uncharacterized. Causes are believed to be multifactorial and include both a significant burden of trauma and disease as well as limited access to quality health services, medical equipment, medications and trained health providers.

Competent provision of emergency medical and trauma care has been shown to decrease patient mortality around the world [1, 2]. The World Health Organization has recently identified the strengthening of acute care capacity of health systems in low income countries (LIC) as a key priority [3, 4]. Ethiopia currently ranks 173/187 on the UN Human Development Index [5] and its health care system is challenged in addressing the high demand for acute care services by a lack of healthcare resources including skilled health care providers. In recent years, the Ethiopian Ministry of Health has taken important steps to bolster the quality and capacity of acute emergency care through a variety of efforts including the establishment of a postgraduate training program in Emergency Medicine (EM) and the recognition of EM as a new medical specialty in the country.

Currently, there is a critical paucity of baseline epidemiological data on all-cause early adult ED mortality in LICs overall [3]. To date, a small number of retrospective chart review studies have been conducted in similarly resourced acute care settings in Africa [6–9]; in Ethiopia, a prospective ED mortality study has not been previously conducted. With the advent of EM as a new Ethiopian medical specialty, this data is crucial for understanding ED mortality patterns to inform the development of targeted patient care interventions to reduce ED mortality, and policy making toward the development of acute care capacity in LIC health systems.

The characterization of ‘early’ ED mortality is important as early mortality is a surrogate for illness severity, and identifies a sub-group of patients who are likely to benefit most from timely therapeutic ED interventions. Early ED mortality is defined in this study as death within 72 h of ED presentation, and ‘very early’ ED mortality is defined as death within 6 h. This ‘very early’ mortality group represents the most urgently ill patients likely to benefit from ED care interventions to prevent mortality. Clinical interventions like standardized ED trauma protocols have been previously shown to decrease mortality in high and middle-income countries but have not been well studied in LIC EDs [10].

Therefore, the purpose of this study was to prospectively characterize all-cause early mortality among adult patients presenting to the TASH-ED and to identify predictors of very early ED mortality to better understand which patients and conditions are most likely to benefit from effective early interventions to reduce mortality in the TASH-ED and in Ethiopia overall.

## Methods

### Study design

This was a prospective cross-sectional observational study.

### Setting and participants

The study was conducted at the Tikur Anbessa Specialized Hospital Emergency Department (TASH-ED), a publicly funded tertiary academic teaching hospital affiliated with the Addis Ababa University School of Medicine with an annual ED census of 18,000 patient visits per year. On average, 50 traumatized and/or critically ill patients are seen in the ED per day, and many require emergent or resuscitative care. At the time of this study, the TASH-ED was staffed by EM and off-service residents and interns supported by consultant non-EM faculty.

### Data collection

Data was prospectively collected from all patients who died within 72 h of presentation to the TASH-ED between October 2012 and May 2013. ‘Early ED mortality’ was defined as death within 72 h of ED presentation based on previously published definitions and clinical rationale [11]. A standardized data collection form was designed and used to record post-death data from patients’ medical records, including the ED triage record, medical intake and clinical care notes and the hospital death certificate. Data collected included demographic information and triage information including method of arrival, chief complaint, triage level, method of payment, time of presentation to ED as well as time from ED presentation to death, time of death and location of death. The standard Ethiopian triage system, called the Ethiopian Emergency Severity Index, classifies patients into five categories based on target time to treat (TTT), mechanism of injury and clinical presentation: red (immediate TTT), orange (TTT <10 min), yellow (TTT <60 min), green (TTT <240 min) and blue (dead).

Four Ethiopian research assistants with nursing qualifications and ED nursing experience were trained by study investigators to extract data using a standardized data collection form. They provided 24-h coverage throughout the study period to prospectively capture all deaths occurring within 72 h of ED presentation, both in the ED and on the hospital wards. The study met the requirements for minimal risk and a waiver of consent was granted by the Research Ethics Boards of the University Health Network in Toronto, Canada and the Tikur Anbessa Specialized Hospital in Addis Ababa, Ethiopia.

The medical record documents for all patients enrolled in the study were digitized to enable quality review by study collaborators. At the midway point of data collection, 15 % of charts were randomly selected and reviewed by the principal investigator (CH), an EM physician, to ensure data extraction and transcription accuracy. Completed data extraction forms were entered into a database by a trained research assistant and verified against the digitized records by the principal investigator (CH) to ensure accuracy of data entry and best-possible clinical accuracy of the documented immediate and contributing causes of death on the original death certificates and data collection forms. A second EM physician (LPR) reviewed a random sample of 10 % of the records to ensure clinical agreement on immediate cause of death, with 100 % agreement between the two reviewers.

### Analysis

Descriptive and analytic statistics were calculated using SAS Version 9.3 (Cary, NC, USA). Summary statistics were calculated for all variables. Following this, all patients dead on ED arrival were excluded from subsequent analyses in order to characterize the subset of patients who may have benefitted from early interventions. Immediate cause of death categories were collapsed into clinically meaningful categories to facilitate analysis; for example, kidney failure, liver failure and gastrointestinal bleeds were amalgamated into ‘other medical causes’ and isolated abdominal, chest, spine and genitourinary trauma were collapsed into ‘isolated non-head trauma’. Pearson’s Chi square and Fisher’s exact tests were used to investigate associations between our outcome variables (a) time to death and (b) immediate cause of death and demographic and clinical variables.

Cause of death was then collapsed into trauma and non-trauma causes and the association between demographic and clinical variables and cause of death was investigated. Patients for whom the immediate cause of death was unknown were excluded from this analysis. Again, Pearson’s Chi squared test, and Fisher’s exact tests were used to test for associations between categorical variables. The student’s t test was used to investigate associations between the distribution of continuous variables and time to death and immediate cause of death.

Time from ED presentation to death was then dichotomized into ≤6 h and >6–72 h. This ≤6 h time frame, defined as ‘very early’ ED mortality, was selected for analysis based on the clinical premise that it represents the most severely ill and injured patient group who would likely benefit most from effective early ED interventions. Logistic regression analysis was used to assess the adjusted impact of the following variables on the probability of dying within 6 h of ED presentation: age, gender, cause of death, time of presentation to the ED, mode of transportation to the ED, day of presentation to the ED and symptom duration. We aimed to estimate a parsimonious logistic regression model adhering to the principle of 10 events per variable [12]. Wald’s test was used to assess whether a given level of a categorical predictor variable was different from the reference level.

This study was approved by the Research Ethics Boards of both the University Health Network in Toronto, Canada and the Tikur Anbessa Specialized Hospital in Addis Ababa, Ethiopia.

## Results

Between October 2012 and May 2013, 9956 patients presented to the TASH-ED. Of these, 220 patients died within 72 h of ED presentation. The times from ED presentation to patient death were as follows: dead on arrival (DOA; 15 %), less than one hour (6 %), 1–6 h (21 %), 6–24 h (37 %), 24–72 h (20 %). We collapsed the data into DOA (n = 34) and non-DOA (n = 186) patient groups. The baseline characteristics of both are displayed in Table 1.

**Table 1:** Baseline characteristics of patients who died within 72 h of ED presentation

Characteristic	Sub categories	N (%)^a^	N (%)^a^	N (%)^a^
		Total sample	Dead on arrival	Alive on arrival
		N = 220	N = 34	N = 186
Baseline characteristics				
Age (years)	<30	66 (30.0)	15 (44.1)	51 (27.4)
	30–50	64 (29.1)	12 (35.3)	52 (28.0)
	>50	90 (40.9)	7 (20.6)	83 (44.6)
Gender	Male	135 (61.9)	27 (79.4)	108 (58.7)
	Female	83 (38.1)	7 (20.6)	76 (41.3)
Cause of death	Congestive heart failure	26 (11.8)	4 (11.8)	22 (11.8)
	Traumatic head injury	45 (20.5)	5 (14.7)	40 (21.5)
	Other medical causes	26 (11.8)	3 (8.8)	23 (12.4)
	Polytrauma/non-head trauma	21 (9.6)	4 (11.8)	17 (9.1)
	Respiratory failure	28 (12.7)	0 (0)	28 (15.1)
	Sepsis/infection	37 (16.8)	2 (5.9)	35 (18.8)
	Stroke	15 (6.8)	1 (2.9)	14 (7.5)
	Unknown	22 (10.0)	15 (44.1)	7 (3.8)
Triage level	Red	73 (34.0)	0 (0)	72 (39.3)
	Orange	89 (41.4)	0 (0)	89 (48.6)
	Yellow	20 (9.3)	0 (0)	20 (10.9)
	Green	2 (0.9)	0 (0)	2 (1.1)
	Blue (dead on arrival)	31 (14.4)	34 (100.0)	0 (0)
Method of referral	Referred from clinic or health post/centre	23 (11.2)	1 (3.5)	22 (12.4)
	Referred from hospital (all)	114 (55.3)	11 (37.9)	103 (58.2)
	Self referral	69 (33.5)	17 (58.6)	52 (29.4)
Duration of symptoms	<4 h	53 (25.7)	17 (70.8)	36 (19.8)
	4–24 h	56 (27.2)	4 (16.7)	52 (28.6)
	>24–48 h	15 (7.3)	0 (0)	15 (8.2)
	>48 h–1 week	27 (13.1)	1 (4.2)	26 (14.3)
	>1 week	55 (26.7)	2 (8.3)	53 (29.1)
Mode of transportation to ED	Ambulance	45 (21.5)	6 (19.4)	39 (21.9)
	Car/truck	23 (11.0)	4 (12.9)	19 (10.7)
	Taxi	130 (62.2)	20 (64.5)	110 (61.8)
	Walk-in/carried	11 (5.0)	1 (3.2)	10 (5.6)
# Prior ED visits in past 30 days	0	179 (83.3)	28 (87.4)	151 (82.5)
	1	19 (8.8)	2 (6.3)	17 (9.3)
	≥2	17 (7.9)	2 (6.3)	15 (8.2)
Day of ED presentation	Weekday	149 (68.7)	24 (72.7)	125 (67.9)
	Weekend	68 (31.3)	9 (27.3)	59 (32.1)
Type of Payment	Free	35 (16.0)	3 (9.1)	32 (17.3)
	Non-free (self-pay)	183 (83.9)	30 (90.9)	153 (82.7)
Timing and location of death				
Time of presentation to ED	Daytime	114 (52.5)	21 (61.7)	93 (50.8)
	Evening	45 (20.7)	5 (14.7)	40 (21.9)
	Overnight	58 (26.7)	8 (23.6)	50 (27.3)
Time of death	Daytime	87 (40.1)	21 (61.8)	66 (36.1)
	Evening	54 (24.9)	5 (14.7)	49 (26.8)
	Overnight	76 (35.0)	8 (23.5)	68 (37.1)
Location of death	In the ED	204 (94)	34 (100)	170 (92.9)
	On the ward (any)	13 (6)	0 (0)	13 (7.1)

^a^Cell totals may vary slightly due to missing data

The overall early ED mortality rate among patients alive on arrival to the ED was 1.9 %. The average age of death was 43.1 years, with a male: female ratio of 1.4:1. Head injury (21.5 %) and sepsis (18.8 %) were the most common causes of death, followed by respiratory failure (15.1 %). Cancer, rheumatic heart disease, tuberculosis and HIV/AIDS were common comorbidities. The majority of patients were triaged as level ‘orange’ (48.6 %) while a minority (n = 22; 12 %) were triaged as ‘yellow’ or ‘green’, the less acute categories on the Ethiopian Emergent Severity Index.

More than 70 % of patients arrived directly from other hospitals, clinics and/or health posts. Approximately 21.5 % of patients arrived by ambulance. Most (82.5 %) were visiting an ED for the first time in 30 days and paid out of pocket for their care (82.7 %).

The majority of patients presented to the ED on weekdays (67.9 %) during daytime hours (50.8 %). The times of patient deaths were evenly distributed between daytime (36.1 %), evening (26.8 %) and overnight (37.1 %) throughout the week. The vast majority of patients died in the ED (92.9 %), including those who died close to 72 h after ED admission.

Associations between demographic and clinical characteristics and traumatic and non-traumatic (medical) immediate causes of death are displayed in Table 2. The mean age of trauma patients (40.35 years, SD 17.92 years) trended towards being lower than that of non-trauma patients (45.16 years, SD 18.09 years, p = 0.09). Overall, relative to medical patients, trauma patients were more likely to be male (p < 0.01), less likely to have had prior recent ED visits (p < 0.01) and were triaged as higher acuity (p = 0.04).

**Table 2:** Association between demographic and clinical characteristics and cause of death, collapsed into trauma versus non-trauma

Characteristic	Sub categories	Trauma	Non-trauma	P value
		N (%)^a^	N (%)^a^	
		57 (31.8 %)	122 (68.2 %)	
Baseline characteristics				
Age (years)	<30	24 (42.1)	27 (22.1)	0.02
	30–50	12 (21.1)	38 (31.2)	
	>50	21 (26.8)	57 (46.7)	
Gender	Male	42 (76.4)	61 (50.0)	<0.01
	Female	13 (23.6)	61 (50.0)	
Triage level	Red	29 (51.8)	39 (32.5)	0.03
	Orange	25 (44.6)	63 (52.5)	
	Yellow	2 (3.6)	16 (13.3)	
	Green	0 (0)	2 (1.7)	
Method of referral	Referred from hospital, clinic, health centre	45 (83.3)	74 (63.3)	0.01
	Self referral	9 (16.7)	43 (36.7)	
Duration of symptoms	<4 h	24 (42.1)	12 (10.1)	<0.01
	4–24 h	24 (42.1)	28 (23.5)	
	>24–48 h	3 (5.3)	11 (9.2)	
	>48 h–1 week	5 (8.8)	20 (16.8)	
	>1 week	1 (1.7)	48 (40.4)	
Mode of transportation to ED	Ambulance	17 (30.4)	20 (17.4)	0.21
	Car/truck	7 (12.5)	12 (10.4)	
	Taxi	30 (53.6)	76 (66.1)	
	Walk-in/carried	2 (3.5)	7 (6.1)	
# Prior ED visits in past 30 days	0	55 (98.2)	89 (74.2)	<0.01
	1	0 (0)	17 (14.2)	
	≥2	1 (1.8)	14 (11.6)	
Day of ED presentation	Weekday	35 (63.6)	85 (69.7)	0.43
	Weekend	20 (36.4)	37 (30.3)	
Type of payment	Free	3 (5.3)	29 (24.0)	<0.01
	Non-free (self-pay)	54 (94.7)	92 (76.0)	
Timing and location of death				
Time of presentation to ED	Daytime	23 (41.1)	65 (54.2)	0.26
	Evening	14 (25.0)	25 (20.8)	
	Overnight	19 (33.9)	30 (25.0)	
Time of death	Daytime	24 (43.6)	40 (33.1)	0.29
	Evening	15 (27.3)	32 (26.5)	
	Overnight	16 (29.1)	49 (40.4)	
Location of death	In the ED	53 (94.6)	110 (91.7)	0.48
	On the ward (any)	3 (5.4)	10 (8.3)	

^a^Excludes DOA patients and those with cause of death unknown, total n = 179

Table 3 shows the association between demographic and clinical characteristics by time to death. The multivariable logistic regression analysis of ED mortality, dichotomized into very early (≤6 h) and later (6–72 h) time to death, revealed the sole significant predictor of death within 6 h to be symptom duration less than 4 h (4–48 h vs. <4 h: OR = 0.20, 95 % CI 0.07, 0.53, p < 0.01; >48 h vs. <4 h: OR = 0.27, 95 % CI 0.09, 0.81, p = 0.02). We conducted post hoc analyses of several different time frames of early mortality (time to death 0–24 h vs. >24–72 h and time to death 0–6 h, >6–24 h and >24–72 h) to investigate whether predictors of time to death other than symptom duration emerged; all analyses yielded similar results.

**Table 3:** Association between demographic and clinical characteristics and time to death

Characteristic	Sub categories	0–6 h	6+ h	P value
		N (%)	N (%)	
		60 (32.3)	126 (67.7)	
Baseline characteristics				
Age (years)	<30	18 (30.0)	33 (26.2)	0.49
	30–50	19 (31.7)	33 (26.2)	
	>50	23 (38.3)	60 (47.6)	
Gender	Male	39 (65.0)	69 (55.7)	0.23
	Female	21 (35.0)	55 (44.3)	
Cause of death	Congestive heart failure	8 (13.3)	14 (11.1)	0.03
	Traumatic head injury	13 (21.7)	27 (21.4)	
	Other medical causes	5 (8.3)	18 (14.3)	
	Polytrauma/non-head trauma	12 (20.0)	5 (4.0)	
	Respiratory failure	6 (10.0)	22 (17.5)	
	Sepsis/Infection	10 (16.7)	25 (19.8)	
	Stroke	3 (5.0)	11 (8.7)	
	Unknown	3 (5.0)	4 (3.2)	
Triage level	Red	38 (63.3)	34 (27.6)	<0.01
	Orange	20 (33.3)	69 (56.1)	
	Yellow	2 (3.3)	18 (14.6)	
	Green	0 (0)	2 (1.6)	
Method of referral	Referred from hospital, clinic, health centre	39 (69.6)	86 (71.1)	0.85
	Self referral	17 (30.4)	35 (28.9)	
Duration of symptoms	<4 h	22 (37.3)	14 (11.4)	<0.01
	4–24 h	12 (20.3)	40 (32.5)	
	>24–48 h	2 (3.4)	13 (10.6)	
	>48 h–1 week	11 (18.6)	15 (12.2)	
	>1 week	12 (20.3)	41 (33.3)	
Mode of transportation to ED	Ambulance	17 (29.8)	22 (18.2)	0.06
	Car/truck	9 (15.8)	10 (8.3)	
	Taxi	27 (47.4)	83 (68.6)	
	Walk-in/carried	4 (7.0)	6 (5.0)	
# Prior ED visits in past 30 days	0	52 (86.7)	99 (80.4)	0.50
	1	5 (8.3)	12 (9.8)	
	≥2	3 (5.0)	12 (9.8)	
Day of ED presentation	Weekday	44 (73.3)	81 (65.3)	0.28
	Weekend	16 (26.7)	43 (34.7)	
Type of Payment	Free	11 (18.3)	21 (16.8)	0.80
	Non-free (self-pay)	49 (81.7)	104 (83.2)	
Timing and location of death				
Time of presentation to ED	Daytime	28 (46.7)	65 (52.9)	0.73
	Evening	14 (23.3)	26 (21.1)	
	Overnight	18 (30.0)	32 (26.0)	
Time of death	Daytime	16 (27.6)	50 (40.0)	0.10
	Evening	21 (36.2)	28 (22.4)	
	Overnight	21 (36.2)	47 (37.6)	
Location of death	In the ED	55 (93.2)	115 (92.7)	0.91
	On the ward (any)	4 (6.8)	9 (7.3)	

Table 4 displays the odds ratios, 95 % CI’s and p values from the multivariable logistic regression model predicting time to ‘very early’ death in the ED.

**Table 4:** Multivariable logistic regression analysis predicting time to death

	Odds ratio	95 % CI	P value
Age <30	–	–	–
Age 30–50	0.71	0.27, 1.90	0.50
Age >50	0.65	0.26, 1.64	0.36
Male	–	–	–
Female	0.90	0.42, 1.90	0.77
Medical cause of death	–	–	–
Traumatic cause of death	1.59	0.64, 3.94	0.31
Sepsis cause of death	1.57	0.56, 4.39	0.39
Daytime presentation	–	–	–
Night-time presentation	0.83	0.38, 1.78	0.63
Transportation, other	–	–	–
Transportation ambulance	1.98	0.84, 4.70	0.12
Weekday presentation	–	–	–
Weekend presentation	0.67	0.31, 1.45	0.31
Symptom duration <4 h	–	–	–
Symptom duration 4–48 h	0.20	0.07, 0.53	<0.01
Symptom duration >48 h	0.27	0.09, 0.81	0.02

Time to death variable dichotomized to ≤6 h and >6–72 h

## Discussion

This study has prospectively characterized the early all-cause ED mortality rate at Tikur Anbessa Specialized Hospital and provides baseline information essential to the development of interventions to address common causes of early ED mortality in Ethiopia.

Our findings of head injury and sepsis as the most common causes of death among ED patients, as well as the gender composition, relatively young average age of death and overall trauma burden among Ethiopian ED patients are consistent with previously reported ED mortality studies in Africa [6, 7, 9, 11, 13–15]. While our all-cause early ED mortality rate of 1.9 % is somewhat lower than those previously reported in similarly resourced settings, it remains higher than those in high income countries with mature EM systems in place and highlights a need for improvements in ED care.

The baseline characteristics of this patient population are very instructive. Starting from the time that patients seek ED care, only one-fifth arrive by ambulance, and most patients arrive as referrals from other health facilities. Taxis are the most common mode of transportation for critically ill and injured patients, which highlights a large unmet need for pre-hospital care services in Ethiopia. Furthermore, fifteen percent of patients in our study were dead on ED arrival. This represents a patient population that is either beyond salvage due to severe traumatic injuries or that presented so late in their course of medical illness that they were unfortunately unable to benefit from resuscitative care.

Among patients alive on arrival, 22 were triaged to lower acuity categories ‘yellow’ and ‘green’, possibly indicating a need for a review of triage guidelines among ED nurses to appropriately expedite care for these critically ill patients. High nursing turnover rates in the ED may contribute to variations in level of triage experience and highlight a need for frequent triage guideline review. Further, nearly one-third of patients presented with symptom duration greater than 1 week, which represents a clinical challenge of managing late presentations of primarily medical illnesses in the ED. However, the majority of patients died within 24 h of ED admission, characterizing an overall population of emergently injured and ill patients with potential to receive the highest benefit from timely ED interventions to reduce their mortality.

The largest volume of critically ill patients presented when the ED is most robustly staffed, on weekdays; however, approximately equal volumes of patients in our study arrived during daytime hours and evening/overnight hours. This suggests that the current ED staffing model could be improved to include more ‘after hours’ coverage by EM residents and faculty. As the cadre of trained EM physicians increases each year and public awareness of the availability of emergency care grows, strategic ED staffing models will need to be continually reassessed to ensure that physician and nursing schedules are designed to meet overall patient volume and acuity demands.

The sole significant predictor of very early mortality (death within 6 h) identified by this study was symptom duration less than 4 h, which is most likely a proxy for severe, acute trauma. Patients with symptom duration less than 4 h tended to be younger and male, and to suffer from either trauma or stroke. Standardized ED care protocols for acute trauma and stroke are not currently in place at TASH-ED, although a trauma registry has recently been established. This ‘very early’ mortality group could benefit from reduced mortality with the implementation of standardized trauma and stroke care protocols. Furthermore, this data underscores an urgent need for public health interventions to reduce the incidence of severe traumatic injuries in Ethiopia, namely road traffic accidents.

The large group of patients that died within 6–24 h of ED presentation highlights an important opportunity for ED care re-organization that could also decrease patient mortality. At present, ED physicians continue to care for patients until a bed becomes available on the wards, which can take several days. If consultant services were requested to assume care responsibility for patients beginning at the time of consultation rather than at the time of bed availability, it would reduce the number of critically ill patients under the active care of ED staff and allow for improved care and decreased mortality among patients requiring intensive care that currently remain in the ED for prolonged periods of time [16]. Emergency department length of stay has been shown to be an independent predictor of hospital mortality in high-income countries, with hospital mortality increasing for every hour increase in ED length of stay among trauma patients [17]. The TASH-ED patient length of stay was not measured in this study but can extend to days, compared with the mean 3.2 h reported in the aforementioned study. In Malawi, a recent pediatric ED study demonstrated a significant mortality reduction from simple interventions including ED care re-organization and may contain some important lessons to adapt to similarly resourced adult ED settings [12].

The primary strength of this study is its prospective design. It provides vital baseline epidemiologic data for monitoring the impact of EM development in the TASH-ED and in Ethiopia overall. The analysis of predictors of very early ED mortality provides key information to guide the development of policy and interventions to improve early ED mortality in this setting.

This study has several limitations, primarily as a result of challenges encountered in capturing accurate mortality data from hospital patient records. First, triage records were at times incomplete regarding contributing causes of death. Vital signs were not universally recorded. Many of these patients arrived in extremis and there may not have been time to complete accurate triage records in real time. However, this inherently limits the medical record data available. Second, death certificate completion was at times illegible, incomplete or clinically inaccurate, accounting for high rates of missing data. As is the case around the world, dedicated training for medical staff responsible for death certificate completion, as well as improved health record charting overall, would improve the accuracy of data capture and enhance data quality available for future research. Finally, no patients in our study underwent autopsy, leaving the ultimate determination of cause of death to be solely clinical. This has been previously shown to inherently limit the accuracy of cause of death determination in ED patients [18]. This challenge is certainly not unique to Ethiopia, and is a common limitation in mortality research.

At the time of this study, the TASH-ED was the only hospital in Ethiopia, and indeed one of a handful in all of Africa, to be staffed with EM postgraduate trainees as the result of a successful postgraduate EM training program at the Addis Ababa School of Medicine. Therefore, it is likely that our prospectively characterized all-cause ED mortality rate of 1.9 % represents a ‘best-case scenario’ and that mortality rates among patients seeking emergency care elsewhere in Ethiopia and in other African EDs are higher, as previously documented. Nevertheless, our study findings are likely generalizable to other tertiary care EDs in Ethiopia and similarly resourced EDs on the African continent.

As a growing number of Ethiopian EM specialists graduate, early ED mortality rates should be expected to fall from this established baseline for multiple reasons: (1) improved quality of clinical care, (2) improved organization of ED care, and (3) increased human health resources available to meet emergent patient health care needs. This study provides findings essential to informing the development of ED clinical care protocols to target high mortality, potentially reversible conditions like sepsis and trauma, and provides comparison data to allow for assessment and monitoring of the impact of ED care initiatives and improvements.

## Conclusion

The mortality burden of trauma and sepsis in the TASH-ED is substantial, and mortality patterns differ between these groups. Trauma patients tend to be male, to be triaged to a higher acuity level on ED presentation, to present with a shorter duration of symptoms, and to suffer a higher burden of very early mortality. Medically ill patients, including those with sepsis, tend to be older, to present with a longer duration of symptoms, to be triaged to lower acuity levels on ED arrival, and to have a longer time to death. As EM develops in Ethiopia, the potential impact of initiatives aimed at reducing mortality among these generally young, previously healthy individuals should be explored, including the development of context-specific ED clinical care protocols, emergency medicine transport systems, trauma prevention advocacy initiatives, EM training programs and changes in hospital culture to foster improved communication, care and flow from the ED to inpatient services.

## Abbreviations

ED: Emergency Department
TASH-ED: Tikur Anbessa Specialized Hospital ED
LIC: low income countries (LIC)
DOA: dead on arrival
HIV/AIDS: human immunodeficiency virus/acquired immunodeficiency syndrome
